# O-Acetyl-GD2 as a Therapeutic Target for Breast Cancer Stem Cells

**DOI:** 10.3389/fimmu.2021.791551

**Published:** 2022-01-03

**Authors:** Jing-Yan Cheng, Jung-Tung Hung, Juway Lin, Fei-Yun Lo, Jing-Rong Huang, Shih-Pin Chiou, Ya-Hui Wang, Ruey-Jen Lin, Jen-Chine Wu, John Yu, Alice L. Yu

**Affiliations:** ^1^ Institute of Stem Cell and Translational Cancer Research, Chang Gung Memorial Hospital at Linkou, Taoyuan, Taiwan; ^2^ Chang Gung Univeristy, Taoyuan, Taiwan; ^3^ Department of Pediatrics, University of California in San Diego, CA, United States; ^4^ Genomics Research Center, Academia Sinica, Taipei, Taiwan

**Keywords:** glycosphingolipid (GSL) glycans, breast cancer stem cells markers, immunotherapy, antibody, PDX (patient-derived xenografts)

## Abstract

**Synopsis:**

A sugar-lipid molecule called OAcGD2 is a novel marker for breast cancer stem cells. Treatment with anti-OAcGD2 mAb8B6 may have superior anticancer efficacy by targeting cancer stem cells, thereby reducing metastasis and recurrence of cancer.

**Background:**

Cancer stem cells (CSCs) that drive tumor progression and disease recurrence are rare subsets of tumor cells. CSCs are relatively resistant to conventional chemotherapy and radiotherapy. Eradication of CSCs is thus essential to achieve durable responses. GD2 was reported to be a CSC marker in human triple-negative breast cancer, and anti-GD2 immunotherapy showed reduced tumor growth in cell lines. Using a specific anti-OAcGD2 antibody, mAb8D6, we set out to determine whether OAcGD2^+^ cells exhibit stem cell properties and mAb8D6 can inhibit tumor growth by targeting OAcGD2^+^CSCs.

**Method:**

OAcGD2 expression in patient-derived xenografts (PDXs) of breast cancer was determined by flow cytometric analyses using mAb8D6. The stemness of OAcGD2^+^ cells isolated by sorting and the effects of mAb8B6 were assessed by CSC growth and mammosphere formation *in vitro* and tumor growth *in vivo* using PDX models.

**Result:**

We found that the OAcGD2 expression levels in six PDXs of various molecular subtypes of breast cancer highly correlated with their previously defined CSC markers in these PDXs. The sorted OAcGD2^+^ cells displayed a greater capacity for mammosphere formation *in vitro* and tumor initiation *in vivo* than OAcGD2^−^ cells. In addition, the majority of OAcGD2^+^ cells were aldehyde dehydrogenase (ALDH^+^) or CD44^hi^CD24^lo^, the known CSC markers in breast cancer. Treatment of PDXs-bearing mice with mAb8B6, but not doxorubicin, suppressed the tumor growth, along with reduced CSCs as assessed by CSC markers and *in vivo* tumorigenicity. *In vitro*, mAb8B6 suppressed proliferation and mammosphere formation and induced apoptosis of OAcGD2^+^ breast cancer cells harvested from PDXs, in a dose-dependent manner. Finally, administration of mAb8B6 *in vivo* dramatically suppressed tumor growth of OAcGD2^+^ breast CSCs (BCSCs) with complete tumor abrogation in 3/6 mice.

**Conclusion:**

OAcGD2 is a novel marker for CSC in various subtypes of breast cancer. Anti-OAcGD2 mAb8B6 directly eradicated OAcGD2^+^ cells and reduced tumor growth in PDX model. Our data demonstrate the potential of mAb8B6 as a promising immunotherapeutic agent to target BCSCs.

## Introduction

Tumors are complex tissues comprising phenotypically and functionally heterogeneous cancer cells (1, 2). One of the pivotal subpopulations in a tumor is cancer stem cells (CSCs), which are highly tumorigenic and chemoresistant (3, 4). CSCs harbor the capacity for self-renewal and differentiation and display resistance to chemotherapy and radiation (5). After treatment with doxorubicin, tumor cells showed increased expression of CSC-like cell surface markers and cytokines, along with increased tumorigenicity *in vitro* and *in vivo* (6, 7). Increased production of cytokines, such as IL-6, IL-8, and MCP-1, and upregulation of transcription factors, including HIF-1α and Stat3, have been observed after treatment with chemotherapeutic agents (8–11). Thus, a great deal of effort has been devoted to the search of clinically relevant biomarkers for better identification and targeting of CSCs.

Ganglioside GD2 has been reported to be a surface marker on CD44^hi^/CD24^lo^ BCSCs in triple-negative human breast cancer cell lines and patient samples (12). Reduction of GD2 expression by *ST8SIA1* (GD3 synthase) knockdown inhibited mammosphere formation and cell motility *in vitro*, completely blocked tumor formation *in vivo*, and changed the CSC phenotype to a non-CSC phenotype (12). In addition, Liang et al. showed that GD2, GD3, and their corresponding biosynthetic enzyme GD2/GM2 synthase maintained a stem cell phenotype in BCSCs (13). Furthermore, GD2 may be associated with cMET to activate the cMET signaling pathway, which in turn induces stem cell characteristics of glioblastoma (14). These findings suggest that GD2 might serve as a marker of BCSCs. However, the anti-GD2 antibody mAb14G2a used in these studies to identify the GD2^+^ cells are known to cross-react with OAcGD2 (15, 16). Thus, it remains unclear whether BCSCs delineated by mAb14G2a is GD2 or OAcGD2.

OAcGD2 is the O-acetyl derivative of GD2 ganglioside. Tumors that express GD2 often concomitantly express OAcGD2 (16). Biological functions of OAcGD2 remain unclear, but O-acetylation is frequently associated with cancer aggressiveness. O-acetylation of GD3 protected glioma cells from apoptosis (17), enhanced their survival, and conferred chemoresistance of leukemia cells (18). In addition, O-acetylation plays an important role in modulating the plasticity of chromatin structure in CSCs by changing the electrical property of acetylated sites of histone and covering up the ubiquitination sites to stabilize many non-histone proteins (19). The presence of OAcGD2 in breast cancer cell lines has been reported (20), but its role in breast cancer and CSCs remains unknown.

In this report, we demonstrated that OAcGD2-positive breast cancer cells displayed characteristic hallmarks of BCSCs. Targeting OAcGD2^+^ BCSCs by a specific antibody triggered apoptosis and hampered mammosphere formation *in vitro* and suppressed the tumor growth *via* reducing BCSCs *in vivo*. These findings suggest that OAcGD2 is not only a new biomarker for BCSCs but also an ideal target for immunotherapy targeting BCSCs.

## Result

### Expression of OAcGD2 in Breast Cancer PDXs

**Figure 1:**
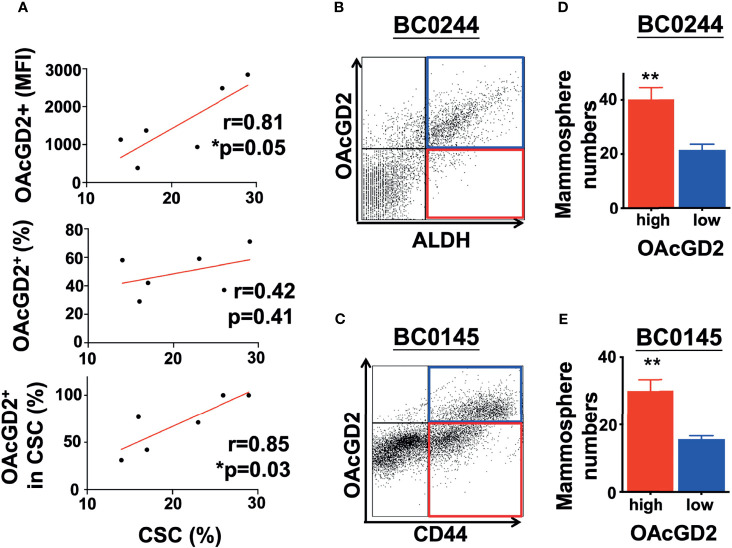
OAcGD2-expressing breast cancer cells harvested from PDXs display enhanced mammosphere forming capacity. **(A)** Pearson correlation analysis between percent of CSCs population and OAcGD2 expression in PDXs as shown in . Pearson correlation coefficient (r) and *p*-value (p) were shown. **(B)** BC0244 cells were stained with anti-OAcGD2 antibody and *ALDEFLUOR*™ kit. The OAcGD2^high^ALDH^+^ and OAcGD2^low^ALDH^+^ BC0244 cells were sorted by FACSAria II and seeded at 1 × 10^3^/well into a 96-well ultra-low attachment plate containing mammosphere growth medium to determine their mammosphere formation ability. **(C)** BC0145 cells were stained with anti-OAcGD2, anti-CD44-APC, and anti-CD24-FITC. OAcGD2^high^CD44^+^CD24^−^ and OAcGD2^low^CD44^+^CD24^−^ BC0145 cells were sorted to examine their mammosphere formation ability. **(D, E)** Seven days after culture, the number of mammosphere was counted under a bright-field microscope. The results were presented as mean ± SD of the mammosphere numbers. Data were analyzed by Pearson correlation analysis and Student’s t-test. *p < 0.05, **p < 0.01.

According to the previous report for OAcGD2 detection by IHC (16), frozen section of the tumor must be used for IHC of OAcGD2 as the deparaffination process may leach out hydrophobic glycolipid molecules such as OAcGD2. Since our original breast cancer specimens are available only as paraffin-embedded tissues, it is difficult to assess OAcGD2 expression in primary tumors. Fortunately, in recent years, the focus of the CSC field has shifted to the use of freshly isolated tumor specimens and early-passage patient-derived xenografts (PDXs), instead of using cultured tumor cell lines (21). Xenotransplantation assays have become an important strategy to assess CSC subpopulations and their activities. We have established five breast cancer PDXs with various molecular subtypes including luminal A and B, triple-negative breast cancer (**Supplemental Table 1**), and identified ALDH as a BCSC marker for BC0244, BC0634, BC0350, VBC108, and CD44^+^CD24^−^ as BCSC marker for BC0145 PDXs (**Table 1**) (22–25). In addition, ALDH is identified as a BCSC marker for PDX AS-B244, which was a subclone of BC0244, designated as AS-B244 (25). Flow cytometry analysis showed that 13–30% of the PDX cells expressed the indicated BCSC markers (**Table 1**). Examination of their expression of OAcGD2 by flow cytometry with anti-OAcGD2, mAb8B6, showed that 30–100% of BCSCs in these six PDXs expressed OAcGD2. Furthermore, Pearson correlation analysis of BCSCs and OAcGD2 expression in these PDXs showed positive correlation of BCSC percentage with OAcGD2 MFI (r=0.8115, *p*=0.05), and percentage of OAcGD2^+^ in BCSCs (r=0.85, *p*=0.03), but not with percentage of OAcGD2^+^ cells (r=0.42, *p*=0.41) (**Figure 1A**). Thus, the amount (MFI) of OAcGD2 is much more pertinent to stemness than its percentage. These findings suggest that OAcGD2 may serve as a marker for further enrichment of BCSCs.

**Table 1:** Expression of OAcGD2 in PDXs of breast cancer.

PDXs	CSC Markers	% of CSCs	% of OAcGD2^+^ Cellsand Expression Level (MFI)	% of OAcGD2^+^ in CSCs
BC0244	ALDH^+^	28–30%	70–73% (2,846)	100%
BC0145	CD44^+^CD24^−^	25–27%	35–40% (2,488)	100%
BC0634	ALDH^+^	15–16%	28–30% (380)	75–78%
BC0350R1	ALDH^+^	20–25%	58–60% (936)	70–72%
BCV108	ALDH^+^	15–18%	40–43% (1,371)	40–43%
AS-B244	ALDH^+^	13–15%	56–59% (1,132)	30–32%

Breast cancer PDXs were stained with anti-CD44-APC, anti-CD24-FITC, or ALDEFLUOR™ kit for detection of BCSCs-enriched subpopulation, as well as with anti-OAcGD2, and analyzed on an EC800 flow cytometer. MFI (mean fluorescence intensity) denotes the intensity of the OAcGD2 expression.

### Expression of OAcGD2 in Breast Cancer Stem Cells

The hallmark of CSCs is their ability to initiate tumors better than their bulk tumor counterparts (26). To determine whether OAcGD2-expressing ALDH^+^ or CD44^+^CD24^−^ BCSCs are more tumorigenic than OAcGD2 negative ALDH^+^ or CD44^+^CD24^−^ BCSCs, we sorted the highest and lowest OAcGD2-expressing ALDH^+^ BC0244 (**Figure 1B**) or CD44^+^CD24^−^ BC0145 cells (**Figure 1C**) to assess their mammosphere-forming ability. The OAcGD2^+^ALDH^+^ BC0244 and OAcGD2^+^CD44^+^CD24^−^ BC0145 cells formed more mammospheres when compared with OAcGD2^−^ALDH^+^ BC0244 (40.2 ± 4.4 vs. 21.5 ± 2.1) and OAcGD2^-^CD44^+^CD24^−^ BC0145 cells (30.0 ± 3.3 *vs.* 15.1 ± 1.7) (*p* < 0.05 for both) (**Figures 1D, E**). To determine the tumor-initiating potentials of OAcGD2^+^ BCSCs, we sorted OAcGD2^low^ and OAcGD2^high^ BCSCs from BC0244 and BC0145 cells and injected these cells into the mammary fat pad of NOD/SCID mice (n = 3/group) at the cell doses of 10^2^, 10^3^, and 10^4^. As shown in **Table 2**, OAcGD2^low^ subpopulation of BC0244 and BC0145 failed to show any tumor engraftment at all three cell dose levels. In contrast, two out of three mice inoculated with 10^3^ or 10^4^ OAcGD2^high^ BC0244 BCSCs grew tumor. Similar results were found in mice injected with OAcGD2^high^ BC0145 BCSCs. These findings support the notion that OAcGD2 is a marker for BCSCs.

**Table 2:** Generation of tumors by OAcGD2^low^ and OAcGD2^high^ breast cancer cells *in vivo*.

	ALDH^+^ BC0244		CD44^+^CD24^−^ BC0145	
	OAcGD2^low^	OAcGD2^high^	OAcGD2^low^	OAcGD2^high^
10^4^	0/3	2/3	0/3	3/3
10^3^	0/3	2/3	0/3	2/3
10^2^	0/3	0/3	0/3	0/3
Frequency	N.D.	1:4,747	N.D.	1:1,072

OAcGD2^−^ and OAcGD2^+^ PDXs cells were sorted by FACSAria II, and 1,000, 100, or 10 cells of the sorted cells were injected into the mammary fat pad of NOD/SCID mice. Tumor formation was observed 8 weeks after transplantation. The frequencies were calculated using Extreme Limiting Dilution Analysis (http://bioinf.wehi.edu.au/software/elda/) based on tumor formation frequency data. N.D., not determined.

### Treatment With mAb8B6 Suppresses Breast Cancer Growth and Reduces Cancer Stem Cells

**Figure 2:**
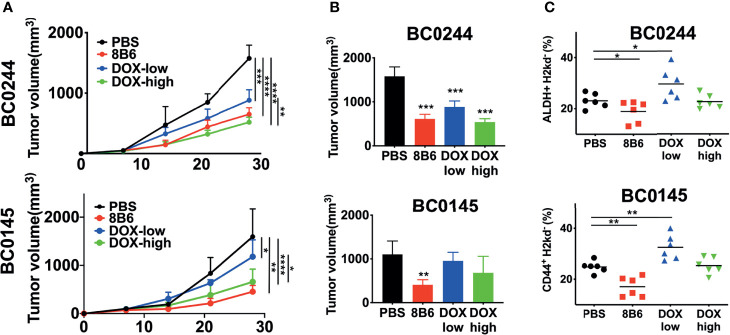
Treatment with mAb8B6 suppresses breast cancer growth and reduces cancer stem cells. **(A)** Growth inhibition of BC0244 and BC0145 PDX tumors in NOD/SCID mice. Mice (n = 6/group) were inoculated with PDX cells (5 × 10^5^ cells) at the mammary fat pad. When the tumor volume reached 100 mm^3^, these mice were intravenously injected with either PBS, 2 mg/kg doxorubicin (DOX-high), 0.5 mg/kg doxorubicin (DOX-low), or mAb8B6 (5 mg/kg) weekly for four times. Tumor volumes were measured every 3 days, and tumor growth was depicted as mean tumor volume for each group. The slope of tumor growth was determined by linear regression. **(B)** Tumor volume was measured before sacrifice (3 days after the last treatment). **(C)** BCSCs frequency in BC0244- and BC0145-derived tumors was identified with *ALDEFLUOR*™ kit and CD44-APC/CD24-FITC, respectively. Data representative of three experiments and values are expressed as mean ± SD and analyzed by one-way ANOVA. *p < 0.05, **p < 0.01, ***p < 0.001, ****p < 0.0001.

Next, we evaluated the antitumor efficacy of mAb8B6, an anti-OAcGD2, on two PDXs of breast cancer, BC0244 and BC0145. Tumor cells were injected into the mammary fat pad of NOD/SCID mice. When the tumor volume reached 100 mm^3^, mice were treated with mAb8B6 (5 mg/Kg), 2 mg/kg of doxorubicin (DOX-high), 0.5 mg/kg of doxorubicin (DOX-low), or PBS once a week for 4 weeks. All animals were sacrificed 3 days after the last administration. As shown in **Figure 2A**, the growth of BC0244 and BC0145 tumors of individual mice and the slope of tumor growth for each group were significantly suppressed by DOX and mAb8B6, with mAb8B6 compared to PBS, *p*<0.0001 in BC0244 and *p*=0.0012 in BC0145. Treatment with DOX showed dose-dependent inhibition-(DOX-high *vs.* DOX-low, *p*=0.0039 in BC0244, *p*=0.0236 in BC0145), and mAb8B6 was as effective as DOX-high in tumor growth inhibition (*p*=0.4195). At the time of sacrifice, the tumor volume of BC0244 in the DOX-low, DOX-high, and mAb8B6 groups was significantly reduced to 56.0 ± 8.6%, 34.1 ± 4.9%, and 38.7 ± 6.5%, respectively, of the control group treated with PBS (*p*<0.001), although the reduction in tumor volume of BC0145 was significant only in the mAb8B6 group (25.8 ± 7.4% of the PBS, *p*=0.0012), but not in the DOX-low (60.5 ± 12.5%), or DOX-high 43.3 ± 23.9% (**Figure 2B**). Thus, based on tumor growth rate or tumor volume harvested after treatment, anti-OAcGD2 treatment significantly suppressed the tumor growth of both PDXs. In addition, we examined the BCSCs (ALDH^+^ for BC0244 and CD44^+^ CD24^−^ for BC0145) in the harvested tumors. BCSCs in both mAb8B6 treated BC0244 and BC0145 tumors decreased to 80.0 ± 17.9% (*p*=0.049) and 68.5 ± 15.6% (*p*=0.0017), respectively, of PBS control group (**Figure 2C**). Although DOX at 2 mg/kg reduced tumor volume, it had no effect on BCSCs when compared to the PBS control group. Interestingly, treatment with 0.5 mg/kg of DOX increased the BCSCs in both BC0145 and BC0244 tumors to 130.9 ± 19.7% (*p*=0.006) and 127.2 ± 26.7% (*p*=0.034) of control group, consistent with the reported relative resistance of BCSCs to chemotherapy.

To further confirm that anti-OAcGD2 treatment can target BCSCs, we tested the frequency of tumor-initiating cells by limiting dilution engraftment assay. BC0244 and BC0145 tumor cells isolated from the mAb8B6- and DOX-treated mice were inoculated into NOD.SCID mice at three different cell doses: 10^4^, 10^5^, and 10^6^. Tumor formation was monitored for 2 months (**Table 3**). As expected, the frequency of tumor-initiating cells in BC0244 tumor treated with mAb8B6 (1:94,752) was significantly lower than those treated with PBS (1:14,241), DOX-low (1:9,100), and DOX-high (1:14,241). Similarly, in BC0145-bearing mice, the frequency of tumor-initiating cells from the mAb8B6 group (1:66,954) was much lower than those treated with PBS (1:14,241), DOX-low (1:5,581), and DOX-high (1:21,636) groups. In line with the increased percent of BCSCs as determined by surface markers, treatment of the BC0244 and BC0145 tumor-bearing mice with 0.5 mg/kg DOX increased the frequency of tumor-initiating cells.

**Table 3:** Estimated frequencies of tumor-initiating cells in PDXs of breast cancer treated with Doxorubicin or mAb8B6.

BC0244: Mice with tumor/total mice				
	PBS	8B6^a^	DOX-low^b^	DOX-high^b^
10^6^	6/6	6/6	6/6	6/6
10^5^	6/6	3/6	6/6	6/6
10^4^	3/6	2/6	4/6	3/6
Frequency	1:14,241	1:94,752	1:9,100	1:14,241
				
**BC0145: Mice with tumor/total mice**				
	PBS	8B6^a^	DOX-low^b^	DOX-high^b^
10^6^	6/6	6/6	6/6	6/6
10^5^	6/6	4/6	6/6	6/6
10^4^	3/6	2/6	5/6	2/6
Frequency	1:14,241	1:66,954	1:5,581	1:21,636

The frequencies were calculated using Extreme Limiting Dilution Analysis.

(http://bioinf.wehi.edu.au/software/elda/) based on tumor formation frequency data.

a 5mg/kg 8B6.

b 2 mg/kg doxorubicin (DOX-high), 0.5 mg/kg doxorubicin (DOX-low).

### Anti-OAcGD2 Treatment Inhibits Proliferation and Mammosphere Formation and Induces Apoptosis of BCSCs *In Vitro*

**Figure 3:**
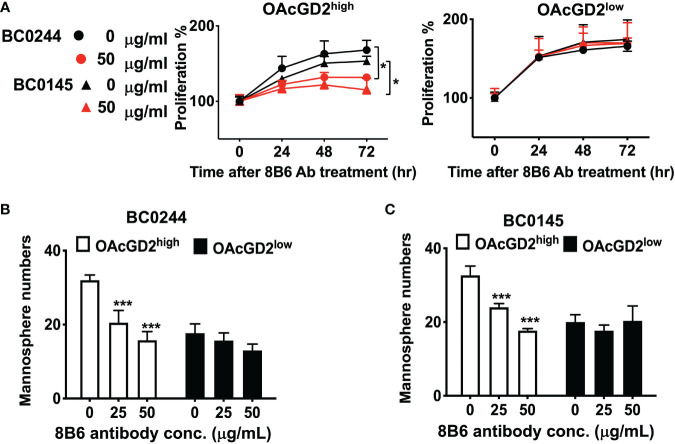
Anti-OAcGD2 mAb8B6 inhibits mammosphere formation and proliferation of PDX cells. **(A)** OAcGD2^high^ALDH^+^ BC0244, OAcGD2^high^CD44^+^CD24^−^ BC0145 cells, OAcGD2^low^ALDH^+^ BC0244, and OAcGD2^low^CD44^+^CD24^−^ BC0145 cells were sorted and treated for 72 h with the indicated concentrations of mAb8B6. Cell proliferation was assessed by the AlamarBlue assay. Optical density was recorded at 570 nm and was expressed as proliferation % normalized to time 0 hr. **(B)** OAcGD2^low^ALDH^+^ and OAcGD2^low^ALDH^+^ BC0244 cells and **(C)** OAcGD2^low^CD44^+^CD24^−^ and OAcGD2^low^CD44^+^CD24^−^ BC0145 cells were sorted and plated at 1 × 10^3^/well in 96-well ultra-low attachment plates containing mammosphere growth medium. Cells were incubated for 7 days with the indicated concentrations of mAb8B6, and the number of mammosphere was counted under a light microscope. The data are presented as the mean ± SD of three independent experiments, each in triplicate. *** *p* < 0.001 compared to cells without 8B6 treatment. *p < 0.05 by Student’s t-test.

We further investigate the *in vitro* effect of the anti-OAcGD2 on the proliferation of OAcGD2^high^ and OAcGD2^low^ BC0145/BC0244, as determined by AlamarBlue assay. As shown in **Figure 3A**, there was no difference in the proliferation rate between OAcGD2^high^ and OAcGD2^low^ subpopulations of both PDXs, but treatment with mAb8B6 inhibited proliferation of OAcGD2^high^ BC0145/BC0244 cells only (left panel), not OAcGD2^low^ BC0145/BC0244 cells (right panel). We next examined the effects of mAb8B6 on the properties of BCSCs. Mammosphere-forming capacity of sorted ALDH^+^OAcGD2^low^ and ALDH^+^OAcGD2^high^ BC0244 cells was assessed in the absence/presence of mAb8B6 at 25 or 50μg/ml. The presence of mAb8B6 significantly decreased mammosphere formation of ALDH^+^OAcGD2^+^ in a dose-dependent manner, while only slightly attenuated the mammosphere formation of ALDH^+^OAcGD2^low^ cells (**Figures 3B, C**).

**Figure 4:**
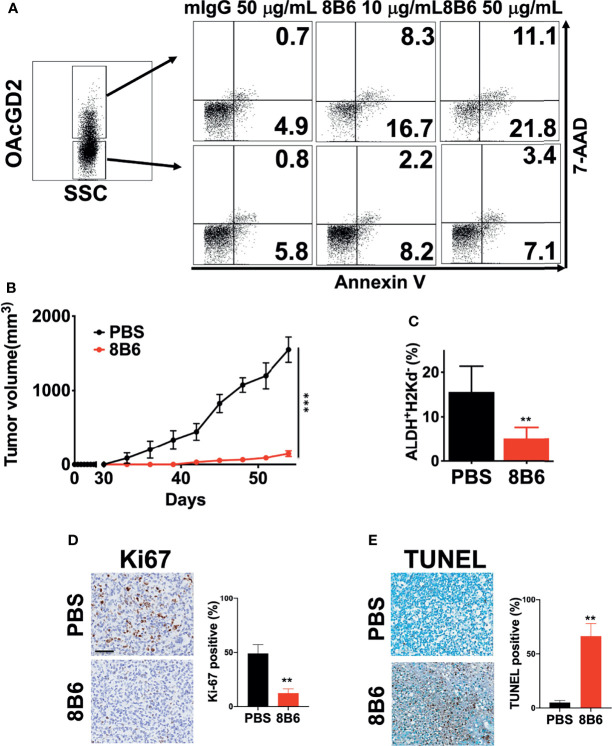
MAb8B6 induces apoptosis in breast cancer cells harvested from PDXs *in vitro* and abrogates tumor growth of OAcGD2-expressing BCSCs in NOD/SCID mice. **(A)** Apoptosis of BC0244 cells after incubation with either 10 or 50 μg/ml of mAb8B6 or mIgG antibody for 24 h was determined by staining with 7-AAD and Annexin V-PE. **(B)** Inhibition of *in vivo* tumor growth of BCSCs sorted from BC0244 in NOD/SCID mice by mAb8B6. Mice (n = 6/group) were inoculated with sorted 1 × 10^5^ OAcGD2^+^ALDH^+^ BC0244 cells at the mammary fat pad. Once the tumor volume reached 100 mm^3^, mice were treated with PBS or mAb8B6 (5 mg/kg) weekly ×4. Tumor volumes were measured every 3 days, and average of the tumor volumes for each group was presented. Tumors were completely abrogated in 3/6 mice treated with mAb8D6. **(C)** Four weeks after tumor inoculation, the BCSCs in BC0244-derived tumors was determined by flow cytometry with *ALDEFLUOR*™ kit and mouse H2K^d^. **(D)** Ki67 and **(E)** TUNEL staining of tumor sections after mAb8B6 treatment. Scale bars, 60 μm. Ki67- and TUNEL-staining-positive cells were counted, and the percentage of positive cells out of the total number of cancer cells was calculated. **p < 0.01, ***p <0.001 by Student’s t-test.

It has been reported that mAb8B6 inhibited the growth of neuroblastoma, small cell lung cancer, and lymphoma cell lines, which was mediated by ADCC/CDC and induction of apoptosis (16, 27). To determine whether mAb8B6 exerts direct cytotoxicity on breast cancer, we examined apoptosis of BC0244 after incubation with mAb8b6 or isotype control antibody for 24 h by flow cytometry. As shown in **Figure 4A**, mAb8B6 induced greater early apoptosis (16.7 and 21.8% at 10 and 50 μg/ml, respectively) and late apoptosis (8.3 and 11.1% at 10 and 50 μg/ml, respectively) of OAcGD2^high^ BC0244 cells as compared to the isotype control antibody (early apoptosis: 4.9%; late apoptosis: 0.7% at 50 μg/ml). On the other hand, mAb8B6 did not induce obvious apoptosis of OAcGD2^low^ BC0244 cells. These results demonstrated the ability of mAb8B6 in inducing programed cell death in OAcGD2-expressing cells.

### Anti-OAcGD2 Treatment *In Vivo* Abrogates Tumor Growth of Isolated BCSCs

**Figure 5:**
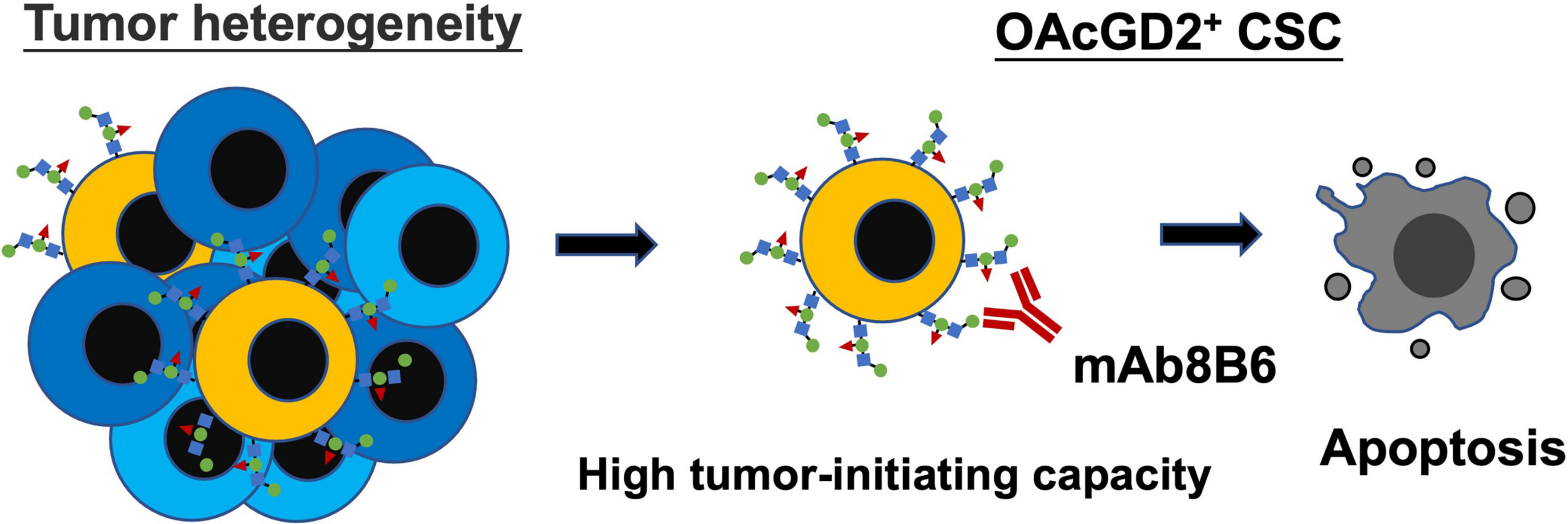
Graphical abstract showing OAcGD2 as a novel marker for CSC which can be targeted with mAb8B6 to suppress tumor growth by inducing apoptosis.

To further ascertain whether mAb8B6 can inhibit tumor growth of OAcGD2-expressing cells *in vivo*, we inoculated 1x10^5^ OAcGD2^high^ BC0244 cells into NOD/SCID mice. When the tumor volume reached 100 mm^3^, mice were randomly divided into two groups for treatment with mAb8B6, or PBS control every week by i.v. injection. It is noteworthy that 50% of mAb8B6 (3 of 6) were completely tumor-free. At 4 weeks, tumors of the remaining three mice of the mAb8B6 group were reduced to 9.4 ± 2.5% (*p*<0.001) of PBS control (**Figure 4B**). Moreover, the remaining tumors from mice treated with mAb8B6 contained significantly less ALDH^+^ BCSCs when compared to those treated with PBS (33.3 ± 15.7% of PBS control, p=0.009) (**Figure 4C**). ﻿To determine whether the anti-proliferative and apoptotic activities of mAb8B6 observed *in vitro* is mimicked *in vivo*, we examined the percentage of Ki67^+^ cells in harvested tumors (**Figure 4D**). The Ki67^+^ cell of tumors obtained from mice treated with mAb8B6 was 12.3± 4.0%, which was significantly lower than the PBS control group (49.8 ± 8.2%, p = 0.002). TUNEL-staining revealed extremely low levels of apoptosis in the tumors from PBS-treated mice (5.0 ± 2.0%) (**Figure 4E**). The percentage of apoptotic cells was significantly higher in the tumors from mice treated with mAb8B6 (66.3 ± 11.6%; p = 0.002). These findings indicate that mAb8B6 can target CSCs by inducing apoptosis and suppress tumor growth as illustrated in **Figure 5**.

## Discussion

In this study, we identified OAcGD2 as a marker for BCSCs. Specifically, OAcGD2 was found to be expressed predominantly on CSCs-enriched population (ALDH^+^ or CD44^+^CD24^−^ cells) harvested from PDXs of different molecular subtypes of breast cancer. Functionally, OAcGD2^+^ CSC demonstrated greater tumor-initiating ability, suggesting their capability for proliferation instead of remaining in a quiescent state. Phenotypically, OAcGD2 expression levels correlated closely with the CSC population in PDX. Our findings provide the first evidence that OAcGD2 is a novel CSC marker for breast cancer. This is in line with the previous report of OAcGD2 as a CSC marker for glioblastoma. The antitumor activity of mAb8B6 against glioblastoma was shown to involve three different mechanisms: (1) induction of antibody-dependent cell cytotoxicity (16), (2) induction of complement cellular cytotoxicity (16), and (3) direct cytotoxicity by inducing pro-apoptosis signal (27). Our results demonstrated that mAb8B6 induced apoptosis of OAcGD2^+^ cells *in vitro*. Treatment of immune-compromised mice bearing PDXs with mAb8B6 *in vivo* resulted in a significant suppression of tumor growth, along with increased apoptotic cells and reduced number of BCSCs. Although NK cell deficit is apparent in NOD-SCID mice, the remnant NK activity may contribute to the observed anticancer effect of mAb8B6 *via* ADCC as reported. These results suggest that anti-OAcGD2 might be an ideal immunotherapeutic agent for BCSCs-targeted therapy of breast cancer.

GD2 has been reported as a specific cell surface marker of BCSCs in triple-negative breast cancer (TNBC) (12, 28–30). In these studies, an anti-GD2 antibody 14G2a was used to identify the GD2^+^ cells. In fact, mAb14G2a has been known to cross-react with OAcGD2 (15). Thus, OAcGD2^+^ cells exhibiting stem cell properties might be included in the cells reactive with mAb14G2a in these reports. On the other hand, mAb8B6 does not cross-react with GD2 (16). Therefore, our studies using mAb8B6 support the notion that OAcGD2 is a bona fide marker for BCSCs and is not limited to TNBC. The amount of OAcGD2 is much more pertinent to stemness than its percentage. The mechanism underlying the contribution of OAcGD2 to CSC properties has yet to be delineated. Currently, the synthetic OAcGD2 is not commercially available, which hampers the progress in this research field. Since the stemness property of GD2 involves HGF-MET (31) and EGFR signaling (28), it may be worthwhile to explore whether these pathways contribute to the stemness property of OAcGD2. In addition, it may be helpful to identify the OAcGD2-binding proteins by immunoprecipitation or OAcGD2-activating genes by RNA-seq. These endeavors may facilitate our understanding of the roles of OAcGD2/mAb8B6 in CSC.

CSCs show functional heterogeneity and hierarchical organization. It is known that CSCs contribute to chemotherapy resistance across a broad range of malignancies (4, 32). Most CSCs are in a quiescent state with a low proliferation rate and thus escape killing by cytotoxic agents that target proliferating cells (33). CSCs possess active drug-efflux machinery, such as ATP-binding cassette family transporters, to pump out chemotherapeutic agents. In addition, overexpression of DNA-repair mechanisms, including homologous recombination, non-homologous end-joining (34), and base-excision repair through increased poly (ADP-ribose) polymerase 1 activity, are very common in CSCs (35). Moreover, CSCs can escape from programmed cell death (36) and acquire an epithelial-to-mesenchymal transition phenotype (37), which facilitate cancer progression and metastasis, respectively. Thus, CSCs have become important targets for cancer treatment. Several therapeutic strategies to target CSCs have emerged, such as the development of a bispecific antibody that brings cytotoxic T cells to CD133^+^ CSCs in pancreatic and hepatic cancers and blockade of CD47 to target CSCs in leukemia. CD47 is a ligand for signal-regulatory protein-α expressed on phagocytic cells and functions to inhibit phagocytosis. Thus, blockade of CD47 has been shown to be an effective strategy for targeting leukemia CSCs in PDX models (38). These CSC-targeting strategies are under clinical development.

Combination of anticancer antibody with chemotherapy is a well-known strategy to enhance the antitumor efficacy. A well-documented chemo-immunotherapy is the combination of CHOP (cyclophosphamide, doxorubicin, vincristine, and prednisone) with Rituximab, which is an effective treatment for aggressive B-cell non-Hodgkin lymphoma (39). Recently, combination of Irinotecan and Temozolomide with an anti-GD2, Dinutuximab, has induced impressive clinical responses in patients with relapsed/refractory neuroblastoma (40). Along this line, preclinical study of the combination of temozolomide and mAb8B6 effectively suppressed the growth of glioma *in vivo* by reducing the temozolomide-resistant stem-like cell pool in glioma (41). This is consistent with our findings of the CSC-targeting capacity of mAb8B6 in breast cancer and suggests that future studies of anti-OAcGD2 in combination with chemotherapy should be explored in breast cancer.

Dinutuximab was approved for the treatment of high-risk neuroblastoma in the setting of minimal residual disease (42) and recently in neuroblastoma patients with refractory/resistant disease (43). However, dinutuximab is associated with dose-limiting neuropathic pain. The lack of allodynic properties of mAb8B6 and abundant expression of OAcGD2 in neuroblastoma (44) make mAb8B6 an attractive option for immunotherapy of OAcGD2-expressing tumors, including neuroblastoma. Future clinical development of mAb8B6 for the treatment of neuroblastoma is warranted. Recent reports have shown that combination of anti-GD2 with PD-1 blockade resulted in synergistic anticancer effects on GD2-expressing tumors in mice, which were attributable to upregulation of immune checkpoint molecules, PD-1/PD-L1, in neuroblastoma by anti-GD2 (45), and induction of immunogenic cell death (submitted manuscript). With the approval of immune checkpoint blockade for the treatment of breast cancer (46), it may be worthwhile to explore whether anti-OAcGD2 may also enhance the anticancer efficacy of anti-PD1/PD-L1 in breast cancer.

In summary, we have demonstrated that OAcGD2 is a marker for CSCs in breast cancer, which can be targeted by mAb8B6 *in vitro* and *in vivo*. Our findings provide strong rationales for the development of anti-OAcGD2 as a novel immunotherapeutic agent for CSC-targeted therapy of breast cancer.

## Materials and Methods

### Cell Culture and Reagent

Human clinical breast cancer specimens were obtained from patients at the time of initial surgery and were fully encoded to protect patient confidentiality. Clinical specimens were utilized under a protocol approved by the Institutional Review Board of the Human Subjects Research Ethics Committee of Academia Sinica, Tri-Service General Hospital, and Veterans General Hospital (Taipei, Taiwan). Isolation of the primary tumor cells from clinical specimens was described previously (24). Five patient-derived xenografts (PDXs) were successfully established from patients BC0145, BC0244, BC0350, and BC0634. BCSC subpopulation was delineated as CD24^−^CD44^+^ cells in BC0145, and ALDH^+^ cells in BC0244, BC350, and BC0634, according to their tumorigenicity (22, 25). All PDXs were maintained throughout xenograft passages. Monolayer cultures of H-2Kd^−^ALDH^+^ BC0244, sorted from xenograft tumors of human primary breast cancer, were designated as AS-B244 cells as described previously (22, 25). The anti-OAcGD2 mAb8B6 is kindly provided by OGD2 Pharma, France.

### FACS Analysis and Sorting

Cell surface OAcGD2 expression on tumor cell lines was assessed by indirect immunofluorescence. Cells were incubated with either mAb8B6 or mouse IgG3 (isotype control antibody) at 10 μg/ml for 30 min at 4°C in 0.1% BSA-PBS. After the reaction, these cells were incubated with the FITC-conjugated goat anti-mouse IgG as a second antibody (Biolegend) for 30 min at 4°C. BCSCs were defined as CD44^+^CD24^−^ or ALDH^+^ cells. ALDH activity was determined by the *ALDEFLUOR*™ kit (Stem Cell Technologies) according to the manufacturer’s instructions. All stained cells were then examined by EC800 flow cytometer (SONY). For sorting, the cells were collected using a BD FACSAria II flow cytometer (BD Biosciences).

### Mammosphere Formation Assay

The sphere culture was performed as previously described (47) with some modifications. OAcGD2^−^ or OAcGD2^+^ CSCs from BC0244 or BC0145 cells were FACS sorted using antibodies against OAcGD2, ALDH, CD44, and CD24. The sorted cells (1x10^3^) were incubated in a mammosphere growth medium in ultra-low-attachment 96-well plates (Corning). All cells grew at a density of 1×10^4^ cells/ml in serum-free Dulbecco’s Modified Eagle’s Medium/F12 supplemented with 20 ng/ml epidermal growth factor, 10 ng/ml basic fibroblast growth factor, 5 µg/ml insulin, 0.4% bovine serum albumin, 100 U/ml Pen/Strep, and 2% B27. Monoclonal antibodies were diluted and added to each well containing 1,000 tumor cells in 96-well plates to give the final concentrations of 0, 25, and 50 μg/ml. After 7 days, the resulting mammospheres were counted.

### Cell Growth Inhibition

Cell viability was measured using the Alamarblue assay. Briefly, sorted cells were incubated with/without mAb8B6 (50 μg/ml) for 72 h at 37°C with 5% CO_2_. Absorbance was measure at 570 nm on a SpectraMAX (M3). The proliferation rate was calculated by normalizing to 0 h.

### Apoptosis

Cells (2×105 cells) were plated in six-well plates for 24 h at 37°C in a humidified atmosphere containing 5% CO_2_, and then treated with 50 μg/ml of mAb8B6 and mIgG for 24 h. After incubation, cells were stained with FITC-conjugated goat anti-mouse IgG (Jackson) as described above. After washing twice with PBS, we resuspended these cells in 500 µl of a binding buffer with Annexin V in the dark for 20 min, according to the manufacturer’s protocol (BD).

### PDXs Tumor Model

NOD/SCID mice were purchased from Jackson Lab and maintained at the animal facility of the Chang Gung University (IACUC number: CGU106-055). Animal studies were conducted by the guidelines for the Care and Use of Laboratory Animals and were approved by the Institutional Animal Care and Use Committee. BC0244 and BC0145 cells (5×10^5^) mixed with 100 µl of 2 mg/ml Matrigel were injected at the base of the nipple of the fourth abdominal fat pad of female mice (4- to 6-week-old). Ear numbering system was used to create a unique identifier; the tumor-free mouse was exclusive. When tumor size reached 100 mm^3^, mice (n = 6/group) were randomly assigned to each group and i.v. injected with 5 mg/kg of mAb8B6, 2 mg/kg of doxorubicin (DOX-high), 0.5 mg/kg of doxorubicin (DOX-low), and PBS once a week for 4 weeks. Sorted OAcGD2^+^ALDH^+^ BC0244 cells (1×10^5^) mixed with 100 µl of 2 mg/ml Matrigel were injected at the base of the nipple of the fourth abdominal fat pad of female mice. When tumor size reached 100 mm^3^, mice (n = 6/group) were randomly assigned to each group and treated with 5 mg/kg of mAb8B6 or PBS once a week for 4 weeks with or without PBMC intraperitoneally at 1×10^7^/mouse as effector cell. Tumor volume was monitored using a vernier caliper twice a week for up to 8 weeks and calculated according to the equation: V = 1/2*W^2^*L, where *L* is the length and *W* the width of a tumor. For ethical considerations, mice had to be euthanized once tumor volume had reached 2,000 mm^3^, which was considered the endpoint for each mouse. In addition, OAcGD2^high^ of ALDH^+^ cells from BC0244 cells and OAcGD2^high^ of CD44^+^CD24^-^ cells from BC0145 cells were sorted using FACSAria II cell sorter (BD) and then inoculated in NOD/SCID mice (n=5). These mice were treated with mAb8B6 or PBS as described above.

### *In Vivo* Tumor Initiation Assay

To obtain single cells from the tumors, we sliced a tumor into square fragments of 1 mm^2^ and then digested these fragments by incubation in a MEM medium containing collagenase (1,000 U/ml), hyaluronidase (300 U/ml), and DNase I (100 μg/ml) at 37°C for 1 h. Single cells (10^4^, 10^5^, or 10^6^) isolated from tumor-bearing mice treated with mAb8B6, DOX-high, DOX-low, or PBS were injected at the mammary fat pad. Animals were examined for tumor formation after 1 week. The frequency of tumorigenic cells and the 95% confidence interval were calculated using Extreme Limiting Dilution Analysis (48).

### ﻿Ki-67 Immunostaining

Tumor tissue sections were deparaffinized followed by antigen retrieval by autoclave for 121°C, 5 min in AR-10 solution (Biogenex). Endogenous peroxidase was quenched before saturating with H_2_O_2_ blocking solution (Dako). Sections were stained with mouse anti-human Ki67 mAb (Leica, Cat No. NCL-L-Ki67-MM1). Bound antibody was detected by polymer-HRP IHC detection system (Biogenex). Digital images were captured by Aperio ScanScope XT Slide Scanner (Aperio Technologies, Vista, CA, USA) under 20× magnification. Positive and negative stained cells were counted on five random fields for each tumor. Data were expressed as cells positive for Ki67 staining/total cells. ﻿

### ﻿TUNEL Immunostaining

The extent of apoptosis in the tumors was measured by TUNEL using the TUNEL assay kit (Abcam, ab206386) following the manufacturer’s protocol. Data were expressed as cells positive for TUNEL staining/total cells.

### Statistical Analysis

Statistical analysis was performed using Prism (GraphPad Software). All values are presented as means ± SD. Three independent experiments were performed, and representative results were shown. * *p* < 0.05, ** *p* < 0.01, *** *p* < 0.001. P-value was calculated by using the Student t-test or one-way ANOVA.

## Data Availability Statement

The original contributions presented in the study are included in the article/**Supplementary Material**. Further inquiries can be directed to the corresponding author.

## Ethics Statement

The studies involving human participants were reviewed and approved by the Institutional Review Board of the Human Subjects Research Ethics Committee of Academia Sinica. The patients/participants provided their written informed consent to participate in this study. The animal study was reviewed and approved by the Institutional Animal Care and Use Committee of Chang Gung University.

## Author Contributions

J-YC, J-TH, and AY conceived and designed the study. JL, J-RH, and S-PC conducted the experiment study. F-YL performed the outcome assessment. Y-HW and R-JL conducted the PDX establishment. J-CW conducted the mice management. J-TH, JY, and AY provided the funding. J-TH, J-YC, and AY analyzed the data and wrote the manuscript with contributions from all authors. All authors contributed to the article and approved the submitted version.

## Funding

This study was supported by grants CMRPG3K2391 from CGMH at Linkou of Taiwan to J-TH, and OMRPG3C0017 and OMRPG3C0018 from CGMH at Linkou of Taiwan to AY, and MOST106-3114-B-182A-001 and 107-2321-B-182A-005 to JY from the Ministry of Science and Technology of Taiwan.

## Conflict of Interest

The authors declare that the research was conducted in the absence of any commercial or financial relationships that could be construed as a potential conflict of interest.

## Publisher’s Note

All claims expressed in this article are solely those of the authors and do not necessarily represent those of their affiliated organizations, or those of the publisher, the editors and the reviewers. Any product that may be evaluated in this article, or claim that may be made by its manufacturer, is not guaranteed or endorsed by the publisher.

## Abbreviations

ALDH, aldehyde dehydrogenase; BCSC, breast cancer stem cell; CSC, cancer stem cell; DOX, doxorubicin, OAcGD2, O-acetly-GD2; PDX, patient-derived xenograft; TNBC, triple-negative breast cancer.
