# Supplementary material for: O-Acetyl-GD2 as a Therapeutic Target for Breast Cancer Stem Cells
Source: Front Immunol. 2022 Jan 3;12:791551. doi: 10.3389/fimmu.2021.791551 (PMC8761789; doi:10.3389/fimmu.2021.791551)
Supplement: Supplementary file 1 [file Table_1.pdf]

Supplementary data
Table 1
Clinical characteristics of PDXs

PDX Code	Diagnosis	Stage	ER	PR	HER2
**BC0145**	IDC	IIIC	−	+	+
**BC0244**	Carcinosarcoma	IIB	−	+	−
**BC0350R1**	Recurrent IDC	III	−	+	+
**BC0634**	IDC	IIIA	−	+	+
**BCV108**	IDC	IIB	−	−	−
**AS-B244**	A subclone established from BC0244				
